# Emulsion electrospinning of sodium alginate/poly(ε-caprolactone) core/shell nanofibers for biomedical applications†

**DOI:** 10.1039/d2na00201a

**Published:** 2022-05-23

**Authors:** Mohammad-Reza Norouzi, Laleh Ghasemi-Mobarakeh, Fabian Itel, Jean Schoeller, Hossein Fashandi, Aurelio Borzi, Antonia Neels, Giuseppino Fortunato, René M. Rossi

**Affiliations:** Empa, Swiss Federal Laboratories for Materials Science and Technology, Laboratory for Biomimetic Membranes and Textiles Lerchenfeldstrasse 5 CH-9014 St. Gallen Switzerland rene.rossi@empa.ch; Department of Textile Engineering, Isfahan University of Technology Isfahan 84156-83111 Iran laleh.ghasemi@cc.iut.ac.ir; ETH Zürich, Department of Health Science and Technology 8092 Zürich Switzerland; Empa, Swiss Federal Laboratories for Materials Science and Technology, Center for X-ray Analytics CH-8600 Dübendorf Switzerland

## Abstract

Electrospun nanofibers have shown great potential as drug vehicles and tissue engineering scaffolds. However, the successful encapsulation of multiple hydrophilic/hydrophobic therapeutic compounds is still challenging. Herein, sodium alginate/poly(ε-caprolactone) core/shell nanofibers were fabricated *via* water-in-oil emulsion electrospinning. The sodium alginate concentration, water-to-oil ratio, and surfactant concentration were optimized for the maximum stability of the emulsion. The results demonstrated that an increasing water-to-oil ratio results in more deviation from Newtonian fluid and leads to a broader distribution of the fibers' diameters. Moreover, increasing poly(ε-caprolactone) concentration increases loss and storage moduli and increases the diameter of the resulting fibers. The nanofibers' characteristics were investigated by scanning electron microscopy, transmission electron microscopy, confocal laser scanning microscopy, Fourier transform infrared spectroscopy, X-ray diffraction, and water contact angle measurements. It was observed that using an emulsion composition of 10% (w/v) PCL and a water-to-oil ratio of 0.1 results in smooth, cylindrical, and uniform core/shell nanofibers with PCL in the shell and ALG in the core. The *in vitro* cell culture study demonstrated the favorable biocompatibility of nanofibers. Overall, this study provides a promising and trustworthy material for biomedical applications.

## Abstract 2

Sodium alginate/poly(ε-caprolactone) core/shell nanofibers were fabricated *via* water-in-oil emulsion electrospinning. The sodium alginate concentration, water-to-oil ratio, and surfactant concentration were optimized for the maximum stability.

## Graphical abstract

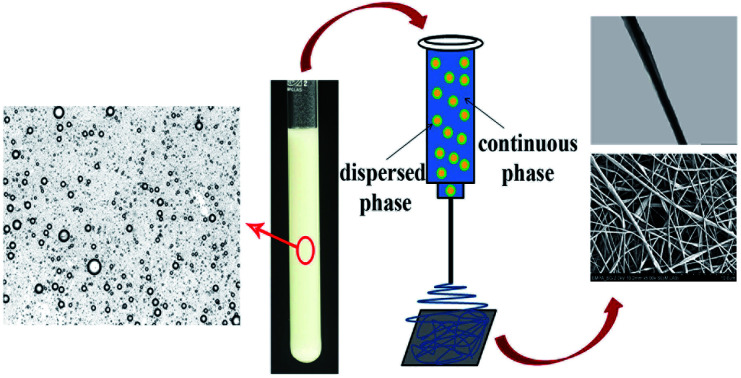

## Introduction

Electrospinning is a robust method to generate polymeric fibers on the nanoscale. During the last few decades, electrospun nanofibers have been extensively studied for different applications due to their inherent properties, such as large surface area and adjustable porosity.^1,2^ These outstanding properties make biocompatible nanofibers ideal candidates for a wide range of biomedical applications.^3^ Nanofibers are especially suitable as tissue regeneration scaffolds due to their structural similarity to the native extracellular matrix, which is well-matched for cell adhesion, proliferation, and differentiation.^4,5^ Likewise, drug delivery is a promising application for electrospun nanofibers due to the high drug loading and encapsulation efficiency of pharmaceutical agents, diversity of polymer selection to achieve compatibility with an intended agent and regulation of release kinetics.^6,7^ Many agents like antibiotics, anticancer drugs, DNA, RNA, and proteins have been successfully incorporated into electrospun fibers to achieve various controlled drug release profiles.^8,9^

Despite recent progress in advanced drug delivery systems, technological challenges exist in encapsulating multiple therapeutic compounds within a single carrier. Two major factors in the design of drug vehicles are versatility and flexibility in drug selection that allow for simultaneous encapsulation of multiple drugs. It is often hard to find a common solvent for agents of different solubility and also a compatible matrix with both hydrophilic and hydrophobic compounds. A clever combination of different agents would enhance therapeutic effects and decrease possible drug resistance. For instance, it has been shown that the co-delivery of lidocaine hydrochloride and mupirocin significantly improved the wound healing process by depositing collagen and completing the re-epithelization.^10,11^

In recent years, due to the versatility of the electrospinning process, some modifications in the conventional electrospinning setup and its parameters were made to achieve fibers with tailored characteristics and morphologies.^12,13^ It has been reported that the core/shell structure is a useful approach capable of protecting sensitive agents during the electrospinning process, controlling the release profile of the incorporated agent, and minimizing the burst release all of which are major limitations in the drug delivery vehicles.^14^ It was stated that the antiangiogenic activity of bevacizumab was preserved after incorporation into poly(ε-caprolactone)-gelatin/poly(vinyl alcohol) core/shell nanofibers and prepared core/shell nanofibers successfully controlled the release of bevacizumab.^15^ Ghazalian *et al.* prepared chitosan/polycaprolactone core/shell nanofibers containing tetracycline hydrochloride drug-loaded core–shell nanofibers showed a two-stage release of the drug, an initial burst release stage followed by a sustained release.^16^

Emulsion electrospinning allows for the production of stable core/shell nanofibers while using a single nozzle spinneret. Compared with coaxial electrospinning, another common method for producing core/shell nanofibers, emulsion electrospinning which is based on single nozzle electrospinning, is more convenient and has greater potential for scale-up.^17,18^ So far, several studies have been carried out on emulsion electrospinning to produce different combinations of polymer matrices with core/shell structures for various applications.^19–22^ A critical factor for emulsion electrospinning is the stability of the emulsions.^18,23^ Emulsions tend to break down over time, and stabilizers such as emulsifiers need to be added to improve stability. Surfactants are the most conventional emulsifiers. These small amphipathic molecules consist of a non-polar tail and a polar head, which adsorb at the oil–water interface. Adsorbed emulsifiers facilitate droplet formation by reducing the interfacial tension and prevent aggregation by increasing repulsive forces.^24^

Poly(ε-caprolactone) (PCL) is an aliphatic polyester widely used in biomedical applications due to its biocompatibility and suitable processing.^25–27^ Several studies have been reported on the electrospinning of PCL.^28–31^ However, encapsulating water-soluble agents in PCL scaffolds is challenging because its hydrophobic nature limits drug–polymer interactions (*e.g.*, hydrogen bonding). Moreover, another problem is the solubility of water-soluble agents in the organic solvents that fit PCL.^32^ Sodium alginate (ALG) is a natural water-soluble biopolymer consisting of mannuronic acid and guluronic acid units in various sequences. ALG has been used extensively in biomedical applications due to its non-toxicity, biocompatibility, biodegradability, and non-immunogenicity properties.^33,34^ ALG-based structured materials are particularly beneficial in encapsulating different agents (drug, cells, and genes) due to the presence of hydroxyl and carboxyl groups in the polymeric backbone allowing for hydrogen bonding and van der Waals interactions with the encapsulated agent.^35^ Furthermore, deprotonated carboxyl groups in glucuronic acid units can be used for simple cross-linking by multivalent cations such as Ca^2+^.^36^ Despite numerous benefits of ALG-based biomaterials, the electrospinning process of ALG has always been challenging. It is due to the polyionic structure and high viscosity of ALG solution, even at very low concentrations. Moreover, ALG is insoluble in the most conventional solvents and water is the only good solvent for it.^37^ Several studies have been conducted to produce ALG-based electrospun nanofibers, mostly using blend and co-axial electrospinning methods.^34,38,39^ However, the emulsion electrospinning ALG has not been considered so much to the best of our knowledge.

The present study aims to prepare stable water-in-oil (w/o) emulsion of ALG/PCL for emulsion electrospinning and produce ALG/PCL core/shell electrospun nanofibers. The prepared electrospun nanofibers would act as a potential candidate for the incorporation of both hydrophilic and hydrophobic pharmaceutical agents. Therefore, several emulsions were prepared by changing the ALG concentration, surfactant concentration, and water-to-oil ratio. The influence of each parameter on the stability of the emulsion was studied. Subsequently, the prepared emulsions were used for electrospinning, and the resulting core/shell nanofibers were characterized. Finally, the biocompatibility of this novel nanofibrous biomaterial was assessed.

## Materials and methods

### Materials

Poly(ε-caprolactone) (PCL, *M*_w_ = 80 000), alginic acid sodium salt from brown algae (ALG, low viscosity), chloroform (CHCl_3_), Span 60 (Span), fluorescein sodium salt (FLS), and Dulbecco's Modified Eagle's Medium (DMEM Sigma-Aldrich) were purchased from Sigma-Aldrich (Switzerland) and used as received.

### Emulsion preparation

The w/o emulsions composed of an aqueous solution of ALG in CHCl_3_ were prepared as follows:

First, an ALG aqueous solution (2, 4, and 6% w/v) was prepared by adding a desired amount of ALG to distilled water. The mixture was magnetically stirred for 4 h until obtaining a clear and homogeneous solution.

Independently, an appropriate amount of Span was dissolved in CHCl_3_; afterward, the aqueous phase of ALG was added dropwise under high mixing speed using a high-shear homogenizer (Kinematica Polytron PT2500 E) at 20 000 rpm for 30 min.

After optimizing the emulsion preparation, PCL was added as the continuous phase to obtain a final PCL concentration of 8, 10, and 12% w/v. The mixture was stirred at 1000 rpm for 4 h to obtain a uniform emulsion.

For the confocal laser scanning microscopy (CLSM, LSM780, Carl Zeiss AG, Switzerland) study, FLS-loaded ALG solution was prepared by dissolving 1 wt% FLS (with respect to the total polymer weight) in the ALG aqueous solution before adding to CHCl_3_.

### Experiment design using the Taguchi method

The experimental design was considered according to the Taguchi approach using Minitab 19 statistical software to optimize the variables affecting the stability of the related emulsion. As shown in Table 1, three control factors, *i.e.* ALG concentration, Span concentration, and water-to-oil volume ratio (*ϕ*_w/o_), were considered to monitor and optimize the stability of the emulsion. Besides, three levels were considered for each of mentioned factors. The stability of new combinations of experimental variables not included in the original experimental design was predicted, and the experimental results were compared with the predicted ones to validate the Taguchi model.

**Table 1:** Experimental parameters and their levels

Parameter	Unit	Level 1	Level 2	Level 3
ALG concentration	w/v%	2%	4%	6%
Span concentration	w/w%	0.5%	1%	2%
*ϕ*_w/o_	—	0.05	0.1	0.2

### Emulsion characterization

The phase separation of the emulsions in test tubes sealed with parafilm and stored at 23 °C was monitored, and images were taken with a digital camera (Nikon D750) at different times. For better observation, the aqueous phase was dyed with FLS in the preparation step, while the oil phase (CHCl_3_) was colorless. The stability of the emulsions was expressed as phase separation percent (PS%), which was calculated as the relation between the heights of the separated aqueous phase (*H*) and the total aqueous phase (*H*_t_) as described in eqn (1). The height measurements were carried out using ImageJ software.

1 PS% = (*H*/*H*_t_) × 100

Light microscopy (Zeiss Axio Observer a1, Carl Zeiss AG, Switzerland) was used to observe the emulsion microstructure and qualitative evaluation. The emulsion drop was deposited on the glass slide, and the installed software captured the image.

The interfacial tension was measured at room temperature (23 °C) by the pendant drop method using Shape Analyzer DSA100 (Kruss, Germany).

Rheological studies were done using an Anton Paar MCR 301 Rheometer equipped with a cone-plate measuring system CP50-2 at 25 °C and 0.2 mm gap. All the measurements were done under a solvent trap to avoid solvent evaporation throughout the experiment. The rotational mode with shear rates in the range of 0.001 to 100 s^−1^ was selected to measure the viscosity of emulsions. In addition, viscoelastic behavior, *i.e.* storage and loss moduli, of the emulsions were studied by oscillatory measurements. Angular frequency sweeps were carried out in the range of 0.1 to 100 rad s^−1^ and applied strain of 0.6%.

### Single nozzle electrospinning

The previously prepared emulsions were placed in a 3 mL plastic syringe (Braun, Germany) fitted with a 23 G metallic needle for electrospinning. The applied potential voltage was 15 kV on the needle and 5 kV on the collector. A syringe pump (KD Scientific, USA) was used to feed the emulsion at a constant rate of 10 μL min^−1^. The fibers were collected on an aluminum foil at a distance of 20 cm from the needle tip.

### Electrospun fibers characterization

The surface of the electrospun fibers was studied by scanning electron microscopy (SEM; Hitachi S-4800, Hitachi High-Technologies, USA & Canada) at an accelerating voltage of 2 kV. The samples were sputter-coated (Leica Microsystems, Switzerland) to increase the electrical conductivity with a 7 nm layer of gold/palladium. The average diameter of fibers was determined based on 50 fibers measurements from the SEM images using the ImageJ software.

Transmission electron microscopy (TEM; Hitachi S-4800, Hitachi High-Technologies, USA & Canada) at an accelerating voltage of 30 kV was used to investigate the structure of electrospun fibers. The sample was prepared by directly depositing a thin layer of fibers on a TEM copper grid throughout the electrospinning process. Additionally, CLSM images of FLS-loaded fibers (excitation: 488 nm and emission: 514 nm) directly electrospun on glass slides were also taken to evaluate the core of the electrospun fibers.

Sodium alginate powder, PCL, and ALG/PCL electrospun fibers were evaluated using Fourier transform infrared spectroscopy (FTIR; BOMEM; Hartmann & Braun, Canada). The FTIR spectra were recorded using the KBr tablet method. The spectra of samples were obtained in the wavenumber range of 400–4000 cm^−1^ with a resolution of 8 cm^−1^.

ALG powders, PCL, and ALG/PCL electrospun fibers were evaluated by X-ray diffraction. XRD diffraction patterns were measured on a Malvern Panalytical X'Pert^3^ MRD instrument using a Bragg–Brentano setup. Data collection was performed at room temperature using Cu-Kα radiation (*λ* = 1.5418 Å) in the range of 2° to 100° in 2*θ*. The angular resolution related to the exploited instrumental setup was 0.05°. Data treatment was performed using the software HighScore Plus for the background subtraction, peak identification, and analysis.^40^

The static water contact angle (WCA) measurement was carried out using Shape Analyzer DSA100 (Kruss, Germany) by the sessile drop method.

### Cytotoxicity assay

The cytotoxicity of the electrospun membranes was assessed by indirect contact tests with normal human dermal fibroblasts (NHDF cells – from a 51 year old person) according to Ulrich *et al.*^41^ The cells were cultured and propagated in 75 cm^2^ cell culture flasks using high glucose DMEM supplemented with 10% fetal bovine serum (FBS), 1% penicillin/streptomycin, and 2 mM l-glutamine at 37 °C in a humidified chamber supplied with 5% CO_2_. The cells were grown until 80–90% confluency, and 3 × 10^6^ cells per flask were used for subcultures.

Extracts of pure PCL, ALG/PCL nanofibers were evaluated. The 6 mm diameter discs were punched out and sterilized under UV light for 30 min each side to prepare the extracts. The discs were then placed in Eppendorf tubes and incubated in 1 mL of cell media without FBS for 24 h and 48 h at 37 °C. Cell media incubated for 24 h and 48 h without sample membranes were used as controls.

NHDF cells were seeded in 96-well plates at a density of 5 × 10^3^ cells per well in cell media and incubated at 37 °C for 24 h. Thereafter, the medium with membrane extracts was replaced with 180 μL of membrane extracts plus 20 μL FBS for 24 h before assessing cell metabolic activity using the PrestoBlue™ assay (LifeTechnologies, Thermo Fisher Scientific, Switzerland). 100 μL of cell media containing 10 vol% of PresotBlue solution was added to each well, following 1 h incubation at 37 °C in 5% CO_2_. 90 μL of the solution from each well was transferred to a new 96-well plate and analyzed using a multimode plate reader (Mithras2 Plate reader, Berthold Technologies, Germany) by measuring the fluorescence intensity (*λ*_exc_ = 560 nm, *λ*_em_ = 590 nm). The values were normalized to cells only. Four independent repeats were performed.

All measurements were made in triplicates and averaged. One-way analysis of variance (ANOVA) was used to statistically evaluate the results. The level of significance was set at 0.05. The data were reported as the mean ± SD.

## Results and discussion

### Preparation of the emulsions

In general, an emulsion is an unstable system that tends to destabilize with time. However, the kinetic stability of the emulsion can be affected by different factors such as the emulsifier structure and concentration, viscosity, volume fraction of the dispersed phase, droplet size distribution, and interfacial properties.^42,43^ In the present study, three factors consisting of ALG concentration, Span concentration, and *ϕ*_w/o_ were considered to study their respective effects on the stability of ALG/PCL emulsions according to the Taguchi method (see ESI†). As shown in Table S1,† the phase separation at the specific time of 45 min (PS_45_) was considered as a response variable in the Taguchi experimental design, as this time is sufficient for electrospinning.

Unlike oil-in-water (o/w) emulsions in which two major factors of steric force and electrostatic repulsion control the stability,^44^ w/o emulsions stability is only governed by steric force because of the low electrical conductivity of the continuous phase. The high mobility of water droplets as a dispersed phase in w/o emulsions usually leads to sedimentation, flocculation, and coalescence. These items act as the reason for lower stability in such emulsions. Increasing the viscosity of the dispersed phase intensifies the hydrodynamic interaction and therefore lowers the sedimentation rate.^45,46^ On the other hand, raising the viscosity increases the size of dispersed phase droplets that enhances the coalescence, and consequently decreases the stability.^43,47^ Hence, a concentration of 4% ALG leading to the highest stability, as demonstrated by the Taguchi experimental design (Fig. S1†), was considered in the following sections.

Six emulsion samples with different Span concentrations and *ϕ*_w/o_ were prepared according to Table 2 while fixing the ALG concentration at 4%. It was observed that except for S_1.0_–ϕ_0.1_, there is an appropriate correlation between the experimental data of PS_45_ and the values predicted with the Taguchi method.

**Table 2:** Experimental and Taguchi-based predicted values of PS_45_ for different emulsions

Samples	Span%	*ϕ*_w/o_	PS_45_ (experimental)	PS_45_ (predicted)
S_0.5_–ϕ_0.2_	0.5%	0.2	39.3%	33.7%
S_0.5_–ϕ_0.1_		0.1	28%	27.9%
S_0.5_–ϕ_0.05_		0.05	19.5%	23.1%
S_1.0_–ϕ_0.2_	1%	0.2	11.7%	13.9%
S_1.0_–ϕ_0.1_		0.1	4%	8.1%
S_1.0_–ϕ_0.05_		0.05	3.8%	3.3%
S_2.0_–ϕ_0.2_	2%	0.2	12.3%	12.9%
S_2.0_–ϕ_0.1_		0.1	5.5%	7.1%
S_2.0_–ϕ_0.05_		0.05	2.3%	2.3%

### Characterization of the emulsions

**Fig. 1:**
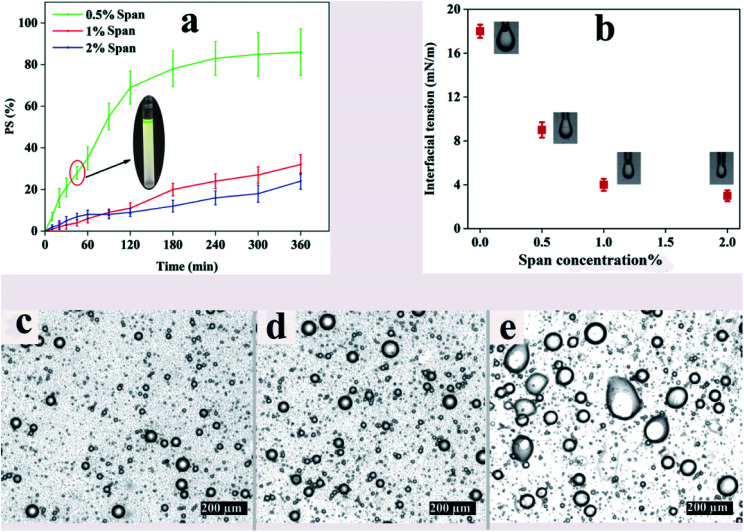
(a) The effect of Span concentration on the phase separation in w/o emulsion (*ϕ*_w/o_ = 0.1), (b) interfacial tension at aqueous ALG–CHCl_3_ interface for different Span concentrations, (c–e) optical microscope images of emulsions after 45 min for different amounts of Span: S_2.0_–ϕ_0.1_, S_1.0_–ϕ_0.1_ and S_0.5_–ϕ_0.1_, respectively.

The phase separation of emulsions with a *ϕ*_w/o_ of 0.1 at different concentrations of Span for a period of 6 h is demonstrated in Fig. 1a. Obviously, all the samples tend to dissociate with time because of the thermodynamic instability of emulsions. In this regard, S_1.0_–ϕ_0.1_ and S_2.0_–ϕ_0.1_ show 32% and 24% phase separation after 6 h, respectively. However, the phase separation in S_0.5_–ϕ_0.1_ is faster than those of the aforementioned samples and after 6 h PS value reaches more than 80%. Illustrated on the chart is a picture of S_0.5_–ϕ_0.1_ at the point of 45 min in which the separated water phase (green phase) at the top of the test tube along with the less opaque oil phase at the bottom is distinct. It is noteworthy that similar trends were also observed for other samples with *ϕ*_w/o_ of 0.05 and 0.2.

Concerning the microstructure of the aforementioned emulsions, light microscopy photography was carried out after 45 min. The resulting images are shown in Fig. 1c–e revealing the coalescence of water droplets in S_0.5_–ϕ_0.1_ in which Span concentration is the least. Thermodynamically, liquid droplets dispersed in an immiscible fluid can quickly coalesce with each other, and therefore the mixture will undergo rapid phase separation which would prevent electrospinning of the emulsions. Interface-active materials like surfactants are usually added to a liquid–liquid mixture to create an energy barrier against droplet coalescence in order to stabilize the emulsion over long periods of time. Adsorption of the surfactant at the liquid interface changes the interfacial properties and alters the surface forces such as steric repulsion between emulsion droplets.^48^ The interfacial tension against surfactant concentration obtained using the pendant drop method is shown in Fig. 1b. It can be seen that the interfacial tension at the ALG–CHCl_3_ interface in the absence of surfactant is about 18 mN m^−1^ while adding 0.5, 1, and 2% Span, decreases it to about 9, 4, and 3 mN m^−1^, respectively.

According to the results, using 1% of Span reduces the interfacial tension between the ALG droplets dispersed in the CHCl_3_, so that the prepared emulsion exhibits good stability, especially within 45 min which is enough for electrospinning. However, a concentration of 0.5% does not provide sufficient stability for electrospinning. Based on the results included in Table 2 and optical microscope images shown in Fig. 1, low stability for all three ratios can be assumed. On the other hand, using Span with a concentration of 2% is not preferable over Span concentration of 1%, as the difference between the interfacial tension values provided by the Span concentrations of 1% and 2% observed in Fig. 1b is not significant, which was further confirmed by the PS_45_ values (Table 2). Taking these results into account, a Span concentration of 1% was regarded to prepare the desired emulsion for electrospinning. In order to make the prepared emulsions spinnable, PCL was dissolved in CHCl_3_ as the continuous phase which can also result in more stability of the emulsion by increasing the viscosity and elasticity of the continuous phase.^49^Table 3 shows the details.

**Table 3:** The specifications of spinnable emulsions prepared for the electrospinning process

Samples	PCL concentration (%)	*ϕ*_w/o_
P_8_–Φ_0.2_	8%	0.2
P_8_–Φ_0.1_	8%	0.1
P_8_–Φ_0.05_	8%	0.05
P_10_–Φ_0.2_	10%	0.2
P_10_–Φ_0.1_	10%	0.1
P_10_–Φ_0.05_	10%	0.05
P_12_–Φ_0.2_	12%	0.2
P_12_–Φ_0.1_	12%	0.1
P_12_–Φ_0.05_	12%	0.05

### Rheological study

**Fig. 2:**
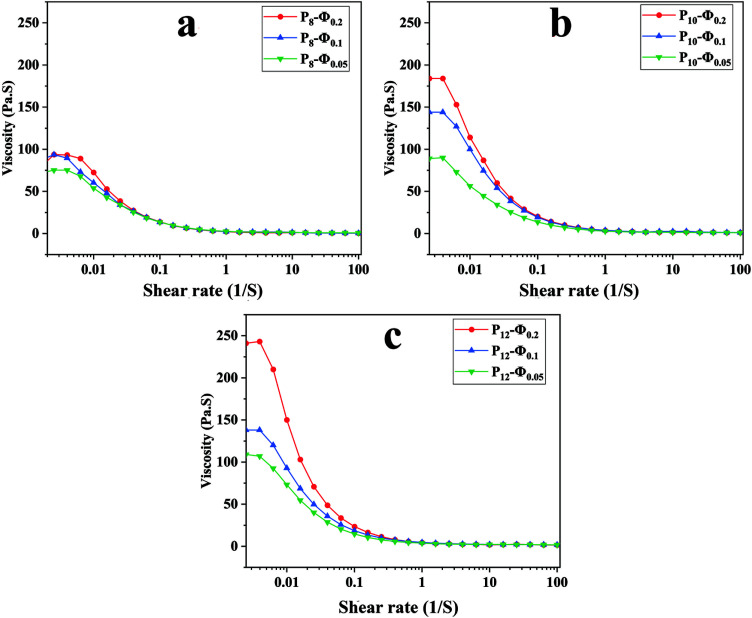
Apparent viscosity against shear rate for ALG/PCL emulsions: (a) PCL 8%, (b) PCL 10%, and (c) PCL 12%.

The apparent viscosity variation of the prepared emulsions against shear rate is depicted in Fig. 2 revealing a shear-thinning behavior for all the samples. An increase in the dispersed phase volume fraction increases the viscosity of the emulsion regardless of the PCL concentration in the continuous phase. This phenomenon could result from larger droplet size and inter-droplet distance at lower *ϕ*_w/o_, which leads to lower viscosity.^50^

To study the deviation of emulsions from a Newtonian fluid and quantify the emulsion's flow behavior, the viscosity and shear rates data were fitted with the Carreau model (eqn (2)).^51^ This model is applied to study the rheological behavior over a wide range of shear rates.^52^

2 [(*μ* − *μ*_∞_)/(*μ*_0_ − *μ*_∞_)] = [1 + (*λγ*)2]^(*n*−1)/2^

where *μ*, *μ*_0_, *μ*_∞_, *n*, and *λ* are the apparent viscosity proportional to shear rate, the Newtonian viscosity at zero shear rate, the Newtonian viscosity at infinite shear rates, the power-law index, and the characteristic time, respectively. The transition point between Newtonian and non-Newtonian regions could be depicted by the 1/*λ* ratio in each sample. Using a shear rate less than 1/*λ*, the fluid will show a Newtonian behavior as a consequence of the amount of the entanglement density.^53^

The Carreau model parameters resulting from the curve-fitting on the emulsion samples data are given in Table 4. According to the table, it is evident that increasing *ϕ*_w/o_ significantly increases *μ*_0_ parameter irrespective of PCL concentration (*p* < 0.05). The same behavior has been recorded for the *μ*_∞_. In addition, the power-law index (*n*) indicates that the higher the *ϕ*_w/o_, the more deviation from a Newtonian fluid. Also, the variation of 1/*λ* against *ϕ*_w/o_ does not show any meaningful trend.

**Table 4:** The Carreau model parameters resulted from the curve-fitting on ALG/PCL emulsions and the mean diameter of resulting electrospun fibers

Samples	*μ*_0_	*μ*_∞_	*n*	1/*λ*	*R*-Squared	Mean diameter (nm)
P_8_–Φ_0.05_	75.8	0.3	0.32	0.000037	0.9984	408 ± 101
P_8_–Φ_0.1_	99.4	0.7	0.13	0.000092	0.9984	358 ± 93
P_8_–Φ_0.2_	164.6	0.8	0.14	0.000039	0.9982	445 ± 286
P_10_–Φ_0.05_	86.8	0.8	0.24	0.000084	0.9993	453 ± 137
P_10_–Φ_0.1_	154.6	1.6	0.13	0.000059	0.9990	364 ± 80
P_10_–Φ_0.2_	201.8	1.5	0.12	0.000042	0.9983	507 ± 319
P_12_–Φ_0.05_	116.8	1.9	0.16	0.000049	0.9984	581 ± 147
P_12_–Φ_0.1_	149.5	2.1	0.14	0.000050	0.9986	525 ± 122
P_12_–Φ_0.2_	296.3	2.2	0.05	0.000032	0.9991	659 ± 288

**Fig. 3:**
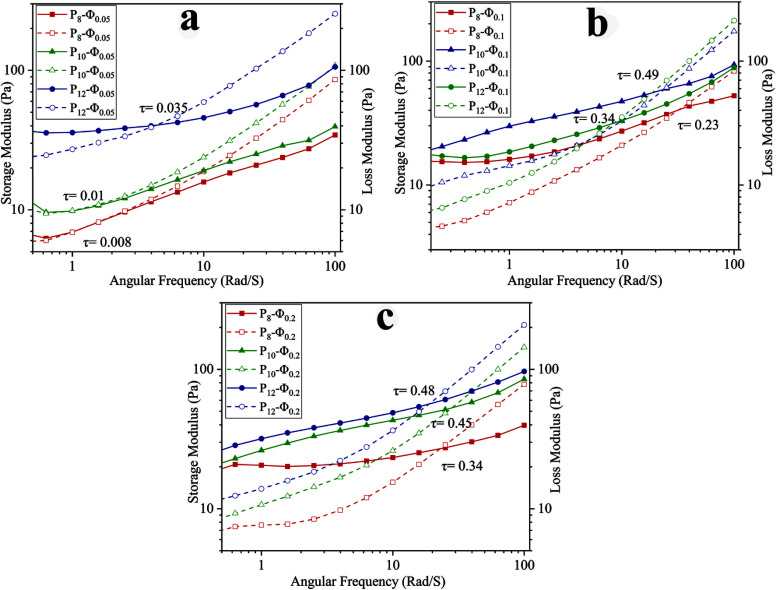
Frequency dependency of *G*′ (solid) and *G*′′ (dashed) for ALG/PCL emulsions: (a) *ϕ*_w/o_ = 0.05, (b) *ϕ*_w/o_ = 0.1, and (b) *ϕ*_w/o_ = 0.2.

To investigate the viscoelasticity of the emulsions when varying PCL concentration and *ϕ*_w/o_, a frequency sweep analysis was conducted. The storage (*G*′) and loss (*G*′′) moduli at different angular frequencies are shown in Fig. 3. At the lower frequency region, *G*′ is higher than *G*′′ and increasing PCL concentration in the continuous phase led to an increase of both *G*′ and *G*′′. The growth in *G*′ value could be associated with caging of dispersed phase droplets as the concentration of PCL in the continuous phase increases.^43^ In other words, after reaching the critical level of PCL concentration, PCL chains not only start to entangle with each other but also arrange an interconnected network around the dispersed phase droplets and this phenomenon is intensified at higher PCL concentration and results in a smaller size for dispersed phase droplet. Furthermore, the growth of the *G*′′ value is due to the presence of more PCL chains in the continuous phase while there is no covalent interaction between them.^43^

In higher frequency regions, for each sample, *G*′′ is higher than *G*′ as shown in Fig. 3. In other words, there is a crossover point above which *G*′′ becomes greater than *G*′ and as the PCL concentration increases, the crossover point occurs at higher shear stress (*τ*). The stress in which the crossover point occurs was calculated based on eqn (3), where *ω*, *t*, and *γ*_0_ are angular frequency, time, and applied strain, respectively.

During the electrospinning process, when the frequency goes beyond the crossover point, viscous behavior becomes dominant. Under this condition, PCL chains in the continuous phase experience higher strain, and as a result dispersed phase droplets start to coalesce with each other along the spinning direction.^43^

3 *τ*(*ω*,*t*) = *G*′*γ*_0_ sin(*ωt*) + *G*′′*γ*_0_ cos(*ωt*)

### Characterization of the electrospun fibers

**Fig. 4:**
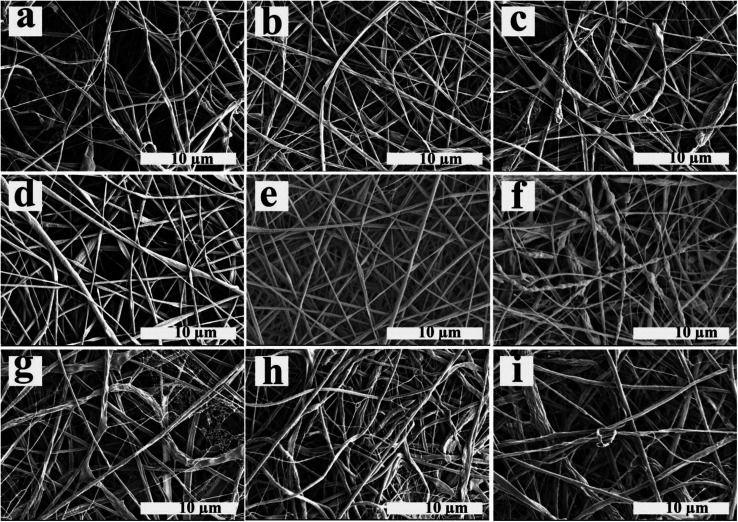
SEM images of electrospun fibers prepared using ALG/PCL emulsions: (a) P_8_–Φ_0.05_, (b) P_8_–Φ_0.1_, (c) P_8_–Φ_0.2_, (d) P_10_–Φ_0.05_, (e) P_10_–Φ_0.1_, (f) P_10_–Φ_0.2_, (g) P_12_–Φ_0.05_, (h) P_12_–Φ_0.1_ and (i) P_12_–Φ_0.2_.

The electrospun nanofibers obtained from emulsion electrospinning of ALG/PCL emulsions were studied and characterized using different methods. SEM images of electrospun fibers are shown in Fig. 4. Furthermore, the nanofibers' diameter distribution histograms are shown in Fig. S2 (see ESI†). As can be observed, bead-free fibers were produced irrespective of the emulsion composition. The concentration of PCL in the continuous phase and *ϕ*_w/o_ are assumed to contribute to the morphology and diameter of the electrospun fibers. Based on Table 4, regardless of the PCL concentration, the standard deviation (SD) at *ϕ*_w/o_ = 0.1 is minimum, implying an improvement in the electrospun fiber uniformity. Furthermore, according to the SEM images, at *ϕ*_w/o_ = 0.2 for all concentrations of PCL, a rough and not-cylindrical structure was observed for the electrospun fibers, which can be attributed to the lower stability of the related emulsion at this ratio (*ϕ*_w/o_ = 0.2) and the possibility of flocculation and coalescence as discussed in the previous section.

At constant *ϕ*_w/o_, the electrospun fibres diameter gradually increased by increasing PCL concentration in the continuous phase. This is explained by the fact that increasing the polymer concentration increases the viscosity of the solution. During the electrospinning process, jet stretching occurs *via* electrostatic and Coulomb repulsion forces, which act against viscoelastic forces. Increasing viscoelastic forces by increasing the viscosity of the polymer solution limits the jet stretching leading to higher fibers diameter.^54^ On the other hand, increasing the chain entanglement as a result of viscosity increment, enable the solvent molecules to be distributed over the entangled polymer chains and result in more uniform and smoother fibers.^55^ In addition, the spider-web effect is detectable at PCL concentration of 12% which is in accordance with previous studies, which confirm that by increasing the polymer concentration, the probability of the spider-web effect rises.^56^ As a result, P_10_–Φ_0.1_ was chosen in order to prepare emulsion-based nanofibers with smooth and cylindrical morphology as well as suitable uniformity.

**Fig. 5:**
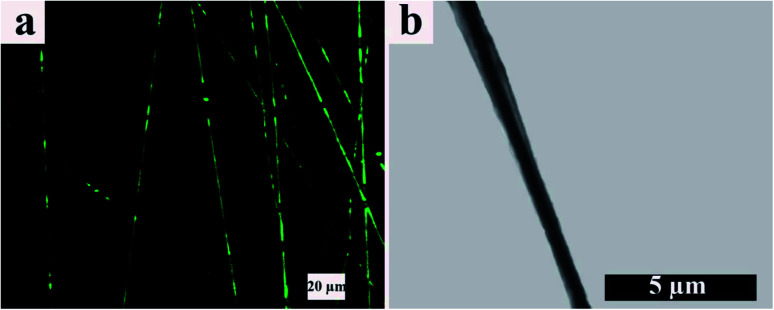
(a) CLSM and (b) TEM images of core/shell electrospun ALG/PCL nanofibers.

TEM and CLSM images of the resulting electrospun fibers are presented in Fig. 5. From the CLSM image (Fig. 5a), it can be observed that ALG, which is located in the core of the electrospun fibers and contains FLS, disappears throughout the fiber axis. In other words, the coalescing process of ALG dispersed droplets is broken up, making FLS undetectable. However, this effect is not predominant within our samples. On the other hand, the TEM image (Fig. 5b) shows a continuous arrangement with a distinct boundary between the core and shell portions. Furthermore, based on TEM, the core/shell boundary moves near the fibers surface at some points, and the shell thickness varies point by point along the fibers axis.

**Fig. 6:**
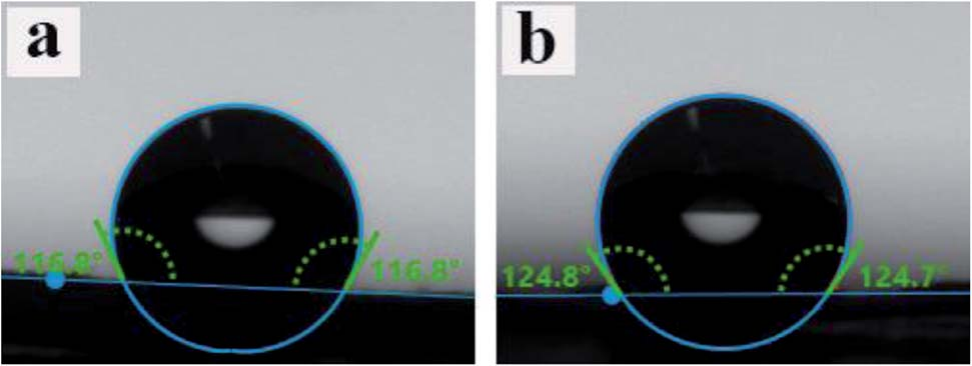
WCA measurement for (a) neat PCL and (b) ALG/PCL electrospun nanofibers.

WCA data for PCL and ALG/PCL electrospun fibers were obtained to evaluate the hydrophobicity of nanofibers' surface. The images are presented in Fig. 6. As can be observed, WCA values for PCL and ALG/PCL nanofibers are about 117° and 125°, respectively, which shows approximately similar hydrophilicity for both ALG/PCL and PCL electrospun fibers. This indicates that PCL is mostly located on the surface of ALG/PCL nanofibers. As discussed in the literature,^57^ during the electrospinning process, CHCl_3_ as the organic solvent in the continuous phase evaporates faster than water in the dispersed phase and hence the viscosity in the continuous phase increases remarkably; so that the outer layer of fibers solidifies faster than the aqueous droplets. This viscosity difference between the organic matrix and the aqueous dispersed droplets leads to an inward movement of the ALG component and the subsequent coalescence. Finally, the hydrophobic PCL forms the shell and dispersed ALG droplets convert to the core of the resulted core/shell electrospun fibers.

**Fig. 7:**
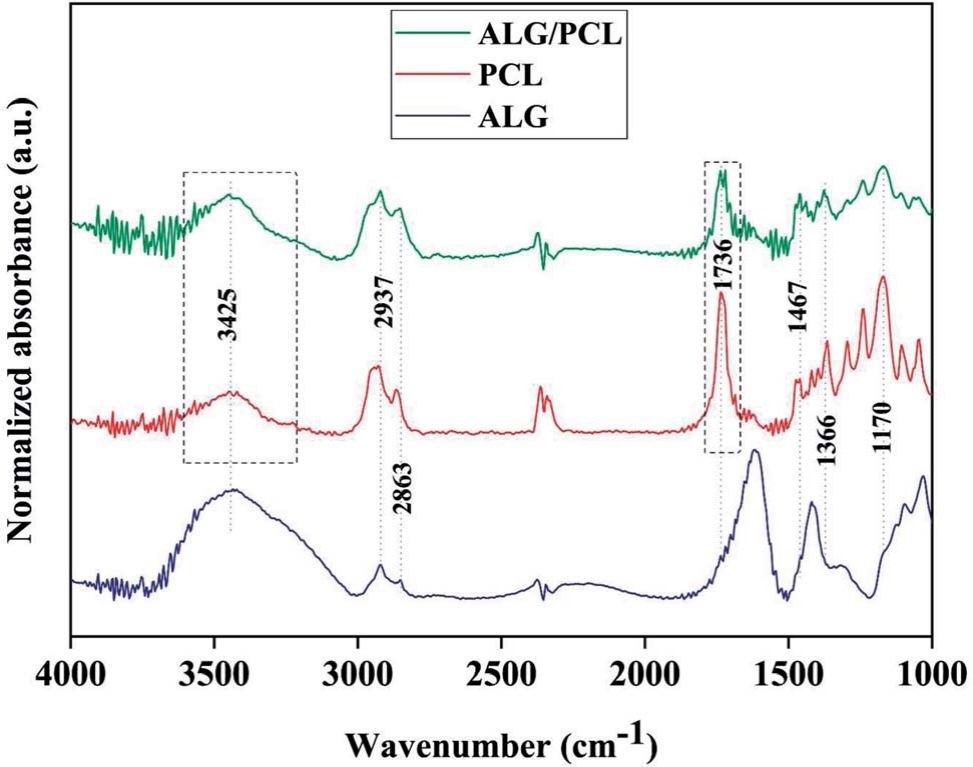
FTIR spectra of ALG powder, PCL nanofibers and ALG/PCL core/shell electrospun fibers.

In order to investigate the chemical composition of core/shell electrospun ALG/PCL fibers, FTIR was carried out. FTIR spectra of ALG powder, PCL, and ALG/PCL electrospun fibers in the wavenumber region of 1000–3800 cm^−1^ are shown in Fig. 7. PCL spectrum displayed characteristic signals at 2937, 2863 (C–H asymmetric and symmetric stretching vibration), 1736 (C=O stretching vibration), 1467, 1366 (C–H bending vibration), and 1170 cm^−1^ (C–O stretching vibration).^58–60^ ALG spectrum showed a broadband at 3425 (hydrogen-bonded O–H stretching vibration), 2937, 2863 (C–H asymmetric and symmetric stretching vibrations), 1615, 1414 (COO^−^ asymmetric and symmetric stretching vibrations), and 1030 cm^−1^ (C–O stretching vibration).^61,62^ Regarding the ALG/PCL spectrum, it is quite similar to the spectrum of PCL electrospun fibers. The height of the broad bond at 3425 cm^−1^ attributed to the hydrogen-bonded O–H stretching, increased in comparison to other signals. This could be explained by the existence of hydroxyl groups in the ALG polymeric backbone. Furthermore, the appearance of the peak at 1736 cm^−1^, which is assigned to the stretching vibration of C=O, has changed from a single peak in PCL nanofibers to a double peak in ALG/PCL nanofibers. Considering the ALG spectrum with no signal in this area, it may be related to hydrogen-bonded carbonyl groups in PCL polymer chains that are located next to the ALG core. This phenomenon leads to the reduced adsorption frequency in this kind of carbonyl group and leads to a double peak signal, one at 1717 cm^−1^ for hydrogen-bonded groups and another at 1736 cm^−1^ for groups without hydrogen bonds.

**Fig. 8:**
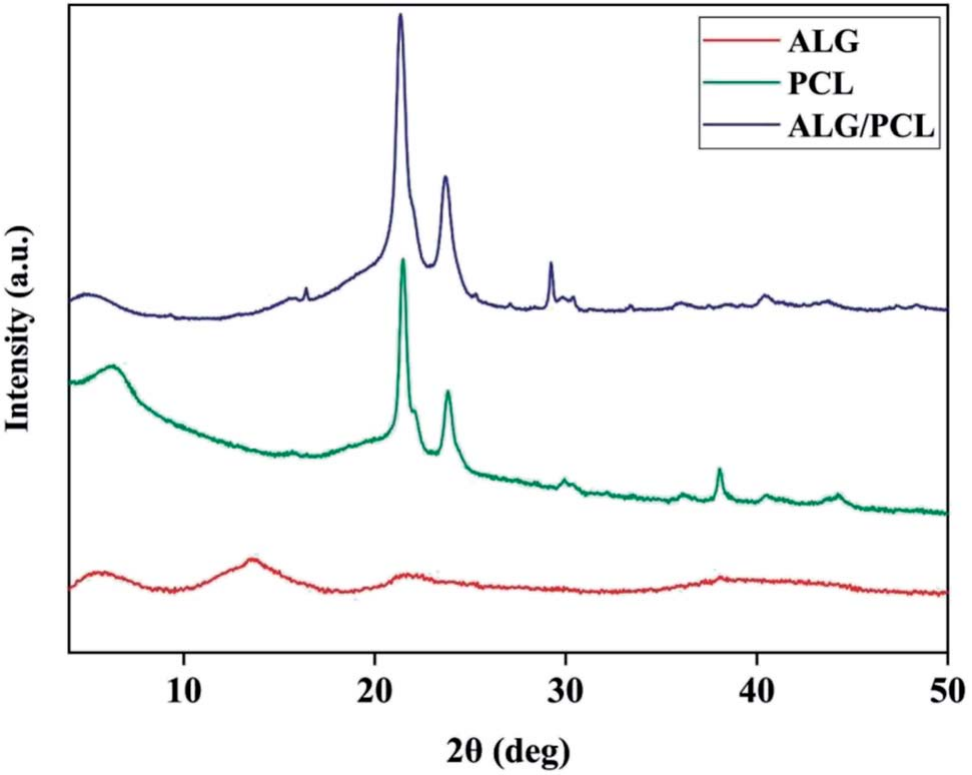
XRD pattern of PCL nanofibers, ALG powder and core/shell ALG/PCL nanofibers.

X-ray diffraction (XRD) was used to characterize the microstructure of nanofibers. The XRD patterns in Fig. 8 show the PCL microstructure as semi-crystalline, with diffraction peaks at 2*θ* angles equal to 21.48° and 23.84°. According to the literature,^63,64^ these peaks are related to the (110) and (200) planes, respectively. The XRD pattern of ALG shows no Bragg's diffraction, which highlights the amorphous microstructure of ALG. As shown by the XRD pattern, ALG/PCL is microstructurally similar to PCL in term of the position of the diffraction peaks. That would be due to the amorphous microstructure of ALG.

Table 5 shows the peaks Full-Width Half-Maximum (FWHM) which was measured using XRD patterns and crystalline domain size of PCL and ALG/PCL electrospun nanofibers. The Scherrer equation^65^ was used to measure the crystalline domain size. Based on the table, incorporation of ALG into electrospun nanofibers decreased the crystalline domain size which means that the structural disorder has been increased. Increasing the structural disorder in the ALG/PCL sample results in the diffraction broadening which leads to an increase in FWHM. So it could be stated that, although the incorporation of ALG as an amorphous polymer did not change the position of the diffraction peaks in PCL, its presence could be approved by increased structural disorder in the ALG/PCL sample comparing PCL.

**Table 5:** The crystalline size and FWHM of PCL and ALG/PCL electrospun nanofibers

Sample	FWHM (°)	Crystalline size (nm)
PCL	0.35	23
ALG/PCL	0.052	15

### Cytotoxicity assay

**Fig. 9:**
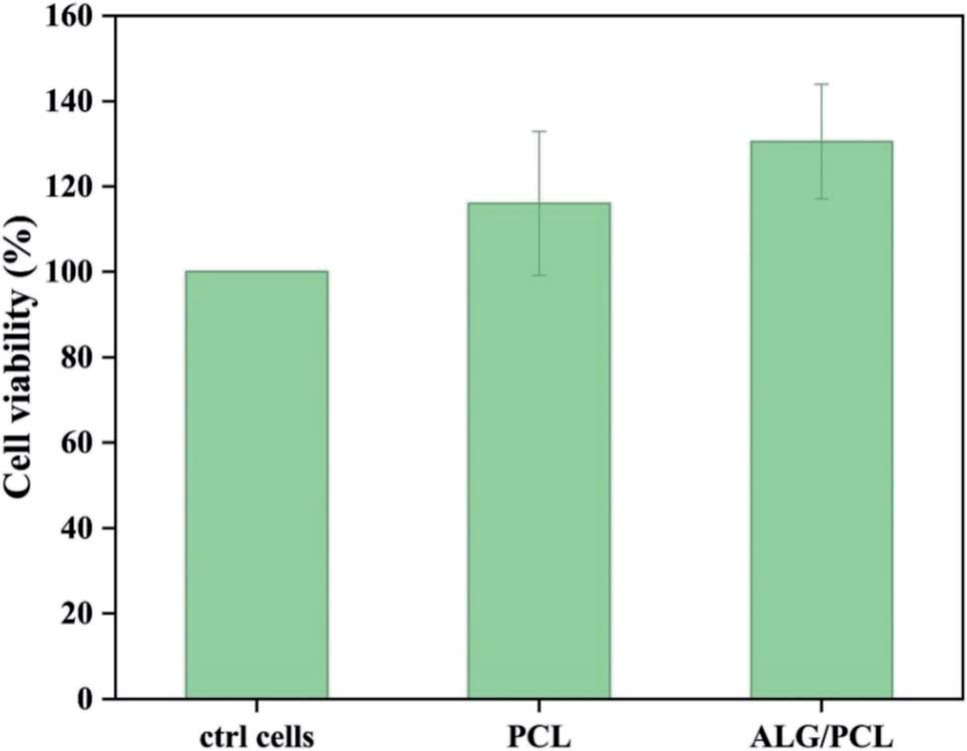
Cell viability of normal human dermal fibroblasts (NHDF) exposed to membrane extracts of PCL and ALG/PCL nanofiber membranes. Values for cell viability were normalized to NHDFs cultured in fresh cell media (ctrl cells). Data are expressed as mean ± sd (*n* = 4).

The cytotoxicity of extracts from PCL and PCL/ALG membranes was evaluated *in vitro* on human dermal fibroblasts (NHDF) (Fig. 9). No cytotoxic effect was observed from sample extracts. The result could be expected as the only possible remaining compound that can be eluted from the membranes is Span, which is known to be a biocompatible emulsifier.

## Conclusions

This work aimed at obtaining core/shell ALG/PCL nanofibers *via* emulsion electrospinning. In this regard, preparation of stable w/o emulsion of aqueous ALG in PCL/CHCl_3_ as the oil phase was considered. The ALG concentration in the dispersed phase and surfactant concentration were optimized regarding the maximum stability of the emulsion. The rheological behavior of the emulsions was discussed using a non-Newtonian fluid model and the viscoelastic characteristic was studied by the oscillatory measurements. Finally, emulsions at different PCL concentrations and *ϕ*_w/o_ were used for emulsion electrospinning. The captured SEM images confirmed homogenous nanofibers with smooth and cylindrical morphology for the electrospun fiber using PCL concentration of 10% and *ϕ*_w/o_ of 0.1. FTIR spectra indicated hydrogen-bond interaction between ALG and PCL in the obtained fibers. Also, TEM and CLSM images revealed the core/shell structure of the electrospun fibers. WCA measurement indicated that PCL is mostly located on the surface of ALG/PCL nanofibers. Moreover, the cytotoxicity assay confirmed no cytotoxic effect for sample extracts. Overall, according to the results, it can be assumed that the designed ALG/PCL core/shell nanofibers have a superior potential to suit biomedical applications such as tissue engineering scaffolds and controlled drug delivery vehicles where simultaneous incorporation and delivery of hydrophilic and hydrophobic agents is demanded.

## Author contributions

M. R. Norouzi conceptualized the study. M. R. Norouzi did project administration; M. R. Norouzi, G. Fortunato and L. Ghasemi-Mobarakeh wrote the project proposal; M. R. Norouzi, F. Itel and J. Schoeller developed the methodology; M. R. Norouzi, F. Itel, A. Borzi and A. Neels performed analysis and interpretation of the results; L. Ghasemi-Mobarakeh, R. M. Rossi and H. Fashandi performed supervision of M. R. Norouzi. M. R. Norouzi wrote the original draft. L. Ghasemi-Mobarakeh, H. Fashandi, F. Itel, J. Schoeller, and R. M. Rossi critically reviewed and edited the manuscript, and M. R. Norouzi revised the manuscript on the basis of these suggestions.

## Conflicts of interest

There are no conflicts to declare.

## Supplementary Material

NA-004-D2NA00201A-s001

NA-004-D2NA00201A-s002

NA-004-D2NA00201A-s003

NA-004-D2NA00201A-s004
