# Supplementary figures and images for: Emulsion electrospinning of sodium alginate/poly(ε-caprolactone) core/shell nanofibers for biomedical applications
Source: Nanoscale Adv. 2022 May 23;4(13):2929–41. doi: 10.1039/d2na00201a (PMC9416811; doi:10.1039/d2na00201a)

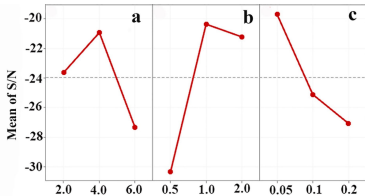

Supplement: NA-004-D2NA00201A-s002 [file NA-004-D2NA00201A-s002.pdf]

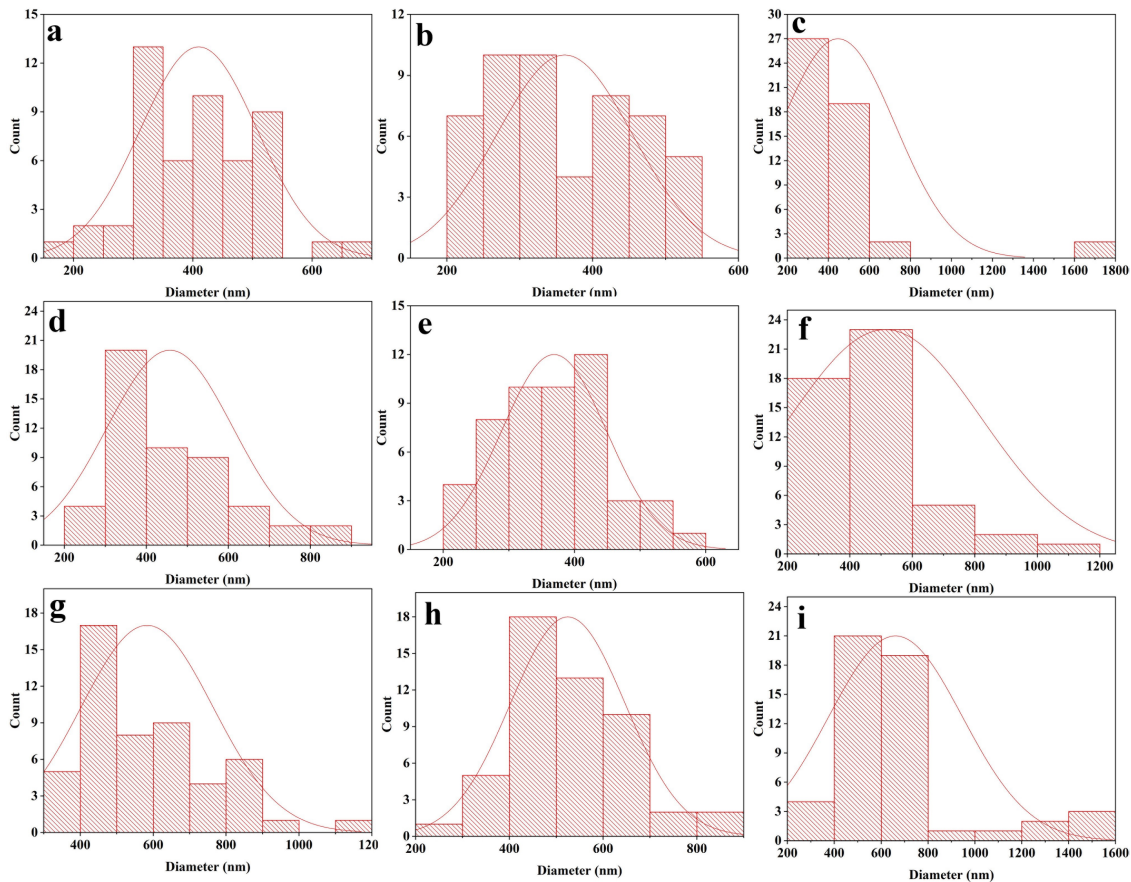

Supplement: NA-004-D2NA00201A-s003 [file NA-004-D2NA00201A-s003.pdf]
