# Supplementary material for: Emulsion electrospinning of sodium alginate/poly(ε-caprolactone) core/shell nanofibers for biomedical applications
Source: Nanoscale Adv. 2022 May 23;4(13):2929–41. doi: 10.1039/d2na00201a (PMC9416811; doi:10.1039/d2na00201a)
Supplement: NA-004-D2NA00201A-s004 [file NA-004-D2NA00201A-s004.pdf]

Electronic Supplementary Material (ESI) for Nanoscale Advances.
This journal is © The Royal Society of Chemistry 2022

Supplementary Information

Table S1. Experimental design with mean PS_{45} and standard deviation (SD)

**Run No.**	**ALG Concentration (w/v)**	**Span Concentration (w/w)**	**$\phi_{w/o}$**	**PS_{45}**	**SD**
1	2%	0.5%	0.2	38.3%	5.8%
2	2%	1%	0.1	12.1%	2.9%
3	2%	2%	0.05	7.1%	1.8%
4	4%	0.5%	0.1	28%	3%
5	4%	1%	0.05	3.8%	1.1%
6	4%	2%	0.2	12.3%	2.6%
7	6%	0.5%	0.05	31.7%	4.3%
8	6%	1%	0.2	23.2%	3.9%
9	6%	2%	0.1	16.7%	2.4%

The Mean signal-to-noise ratio (S/N) diagram for each factor can be seen in Figure S1. We can see that increasing ALG concentration from 2% to 4% leads to a significant increase in S/N, which means higher stability of the emulsion; while a further increase from 4% to 6% decreases the S/N remarkably. Similarly, raising the concentration of Span from 0.5% to 1% increases S/N while adding more Span has no major effect on the stability of the system and S/N does not change considerably. Additionally, the S/N variation trend of the effect of $\phi_{w/o}$ indicates that a higher water to oil ratio, leads to less stability of the emulsion.

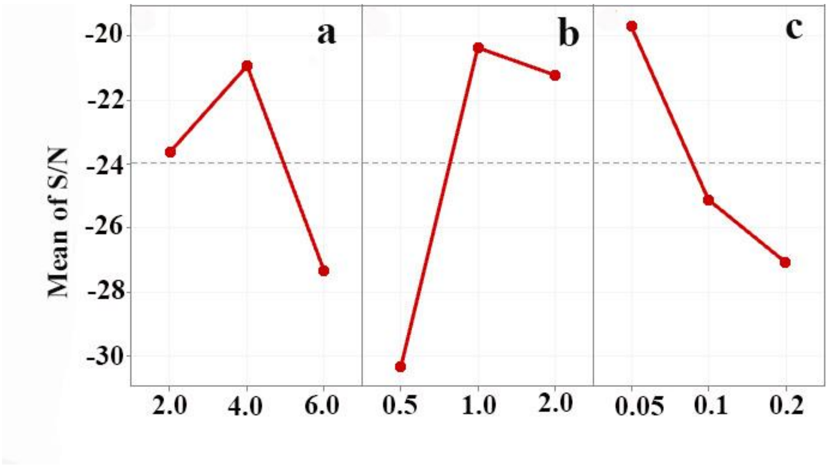

Figure S1. Mean of S/N for: (a) ALG concentration, (b) Span concentration and (c) $\phi_{w/o}$.

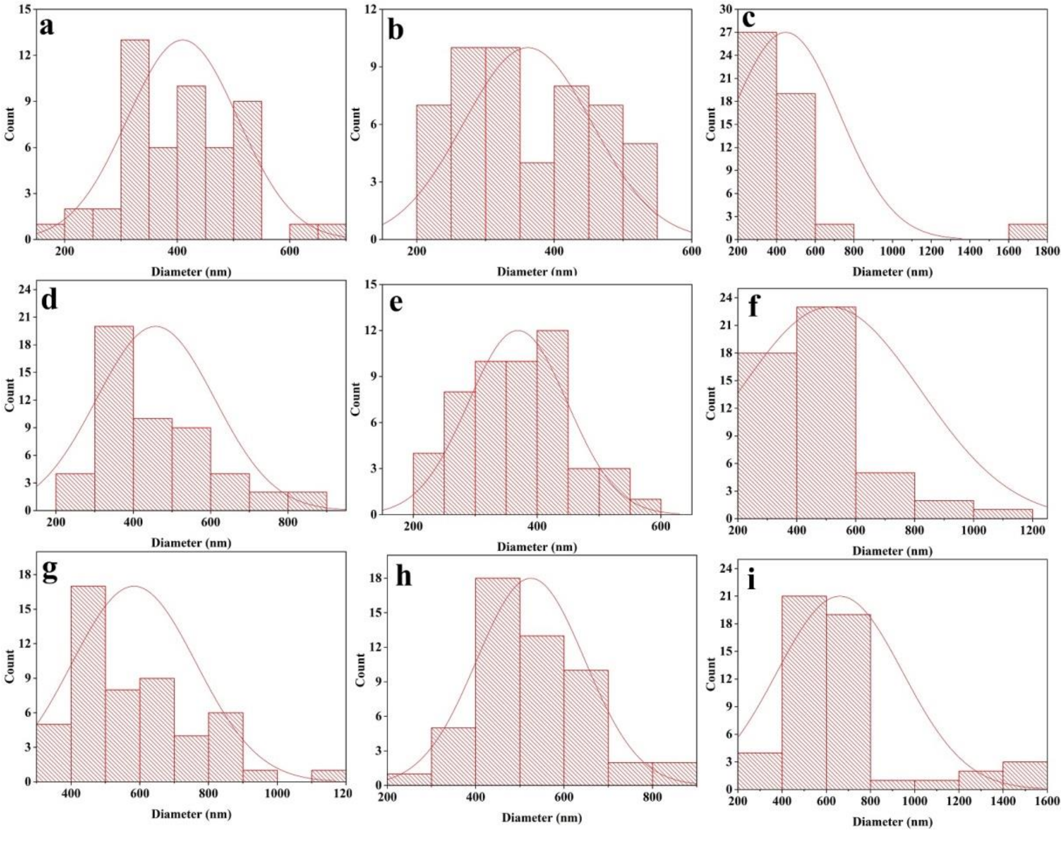

Figure S2. Fiber diameter distribution histograms prepared using ALG/PCL emulsions: (a) P_8-$\Phi_{0.05}$, (b) P_8-$\Phi_{0.1}$, (c) P_8-$\Phi_{0.2}$, (d) P_{10}-$\Phi_{0.05}$, (e) P_{10}-$\Phi_{0.1}$, (f) P_{10}-$\Phi_{0.2}$, (g) P_{12}-$\Phi_{0.05}$, (h) P_{12}-$\Phi_{0.1}$ and (i) P_{12}-$\Phi_{0.2}$.
